# Posture App (version 2.1.66)

**DOI:** 10.5195/jmla.2020.979

**Published:** 2020-07-01

**Authors:** Heather S. Healy

**Affiliations:** 1 heather-healy@uiowa.edu, Clinical Education Librarian, Hardin Library for the Health Sciences, University of Iowa, Iowa City, IA

## Abstract

**Posture App** (version 2.1.66); Muscle&Motion, Tel Aviv, Israel; https://www.muscleandmotion.com/posture/; individual subscriptions: $30/month, discounts for 1- and 3-year subscriptions; contact for institutional pricing: sales@muscleandmotion.com; available for Android, iOS, Mac, and PC.

## PURPOSE

Posture App by Muscle&Motion provides tests, treatment information, and therapeutic exercises for common postural disorders (e.g., kyphosis, lordosis, and flat back). The app also contains muscular, skeletal, and kinesiology anatomy; an electronic book, *Postural Disorders and Musculoskeletal Dysfunction: Diagnosis, Prevention, and Treatment*; core training exercises; and yoga asanas. Apart from the e-book, the content is provided primarily through instructional videos that feature exercises performed by models or illustrative demonstrations. The anatomy sections provide interactive exploration of anatomical structures.

Muscle&Motion, founded in 2001, is headquartered in Tel Aviv, Israel, and has several other apps available for purchase, including the Strength Training App, Anatomy App, and Yoga App. The company's aim is to “enhance an individual's understanding of the muscular mechanics involved in any particular movement of the human body; thereby, improving results, reducing the risk of injury, and providing an overall greater awareness of muscles in motion” [1]. The company website lists Amit G. Alon, company founder, and Gil Solberg, cofounder and author of the included e-book, as the team behind Posture App.

## INTENDED AUDIENCE

The Muscle&Motion website indicates that the product is intended for “teachers, therapists, and instructors of all movement methods” [2]. The developers specifically list fitness trainers; Pilates, dance, and yoga instructors; coaches; chiropractors; physical therapists; occupational therapists; and fitness enthusiasts as targeted users.

The anatomy and exercise content in the app gives it broad appeal to many health sciences disciplines. At this reviewer's institution, a faculty member from the Dance Department requested purchase of the app. Anticipated crossover with faculty of the Health and Human Physiology Department made the purchase appealing. This app and its companion apps may be useful for librarians to be aware of to include on resource lists or to provide answers to reference questions from users who are seeking anatomy or exercise programs.

## CONTENT AND MAJOR FEATURES

The content sections that are available from the Posture App's main menu are New & Popular, Postural Disorders, Therapeutic Exercises, Yoga Asanas, Muscular Anatomy, Kinesiology & Skeletal, and E-book. When opening these sections, content for a subsection is automatically displayed. Users can access other subsections through a sidebar menu by touching a small yellow bubble on the left side of the screen. Much of the content is cross-referenced in other areas and, thus, appears in the menu list for those sections.

The first main section, New & Popular, appears to highlight content segments that are popular with other users of the app or that have been recently added, but no further information is provided regarding these displays. Postural Disorders, the next main section, includes subsections for core training anatomy, kyphosis, lordosis, flat back, general postural tests, kinesiological issues in the movement system, and kinesiological issues in strength training. Each of these subsections includes exercise demonstrations or illustrated videos.

Therapeutic Exercises provides all the exercises in the app that are assigned to categories including core training, manual therapy, muscle lengthening for different body regions, various yoga categories (e.g., backbends, rotation postures), clinic videos, and foam roller exercises. The Yoga Asanas section includes all the yoga exercises and categories provided in the Therapeutic Exercises section as well.

**Figure 1:**
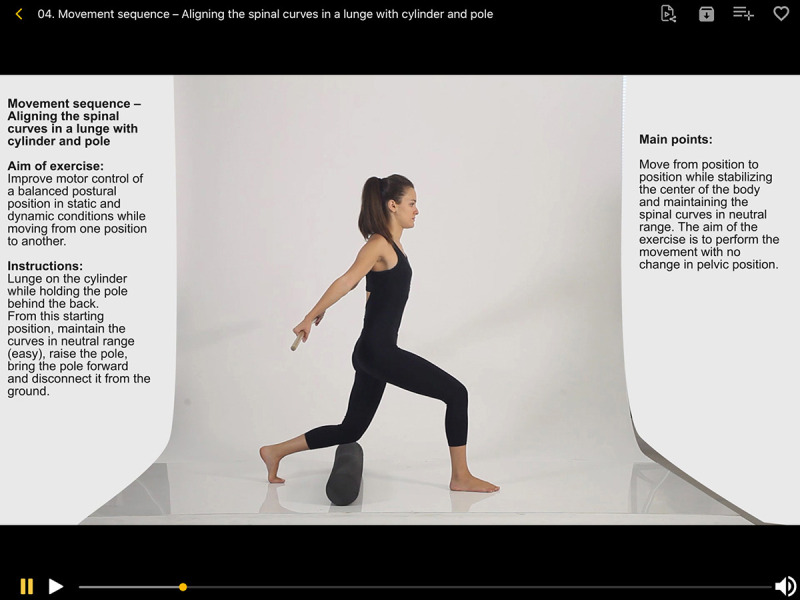
Screenshot of a movement sequence in Posture

The Postural Disorders and Yoga Asanas main sections include a subsection link called Anatomy Trains that offers a collection of videos demonstrating stretching and movement along various lines (e.g., lateral line, superficial front line). These sections as well as the Therapeutic Exercises section also provide an A–Z Theory Videos Index that allows users to browse all videos by name or search for videos on a desired topic (Figure 1).

**Figure 2:**
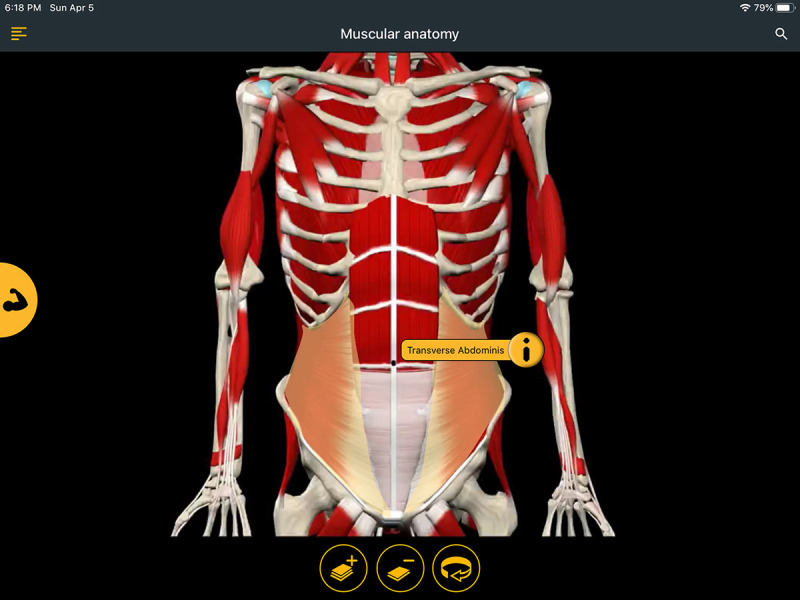
Screenshot of a body image in Posture

The Muscular Anatomy and Kinesiology & Skeletal sections provide an interactive tool for extensive exploration of anatomical structures, with options for removing or adding layers of muscle and rotating the illustrations 360 degrees. Additionally, labels for each structure can be accessed by tapping the area of interest. Touching the “i” icon on any of the labels provides additional information and often more detailed images of the body region (Figure 2).

The e-book, *Postural Disorders and Musculoskeletal Dysfunction: Diagnosis, Prevention, and Treatment,* can be accessed from the app's main menu. The book compromises 14 chapters, for a total of 303 pages of content. The text is easily navigated from the table of contents provided on the sidebar menu. Unlike the rest of the app, the book does not realign when users rotate their devices, and the text is small. Users may need to use pinch-to-zoom gestures for adequate resizing on mobile devices.

## USABILITY

In general, the app is usable and easy to navigate. It can be used within seconds of download and login, but some exploration may be needed to understand the content's organization. As noted, many sections of the app can be accessed from other sections, which led to some initial confusion about the overarching organization. Moreover, the content of the app was often more extensive than anticipated. For example, many of the exercises have multiple pages of videos to swipe through, and the anatomy sections frequently have several levels of content for each body region.

Many of the videos play on a continuous loop, and the user must touch the stop button to end play; however, they also stop when the user moves to another portion of the app. This reviewer did encounter a problem when an unidentified video continued playing repeatedly even as other content was being viewed. The app was reinstalled to resolve this issue.

This reviewer also experienced some difficulty in opening the sidebar menu. The touch sensitive screen area seems to be smaller than the actual bubble that opens the sidebar menu. Sometimes repeated attempts were needed to successfully open the menu.

The exercise videos are easy to follow and provide text, narration, and visual demonstrations. This reviewer found the app useful to add variety to a home exercise routine and for exercises to counteract sitting at a desk for long periods of time.

The app's main menu includes features for My Favorites, My Folders, and My Downloads. The first two allow users to mark various segments of the app's content for quick and easy future access. The My Downloads option provides downloading of content segments—such as tests, exercises, and asanas—for offline use. In addition to any segments saved to My Downloads, the e-book is also available for offline reading. All other portions of the app require an Internet connection.

## PURCHASING OPTIONS AND IMPLEMENTATION

Individual subscriptions are available for $30.00 per month. Reduced prices are available for a 1-year ($7.45 per month) or 3-year ($6.60 per month) rate. See the website for other potential discounts. Universities and colleges can obtain a 1-month free trial. Additionally, free versions (with only portions of the content) are available for all the Muscle&Motion apps.

Institutional pricing is offered at different levels, including 20 simultaneous logins, 100 simultaneous logins, or 300 simultaneous logins. Implementation was simple, and setup only involved creating a username and password. The password will need to be reset at regular intervals, likely on a semester basis, for a library to provide access to the app for new groups of users. At the reviewer's institution, the credentials are made available first for students in courses using the app and then to users who request them.

## CONCLUSION

The format and ease of portability on a mobile device makes this a useful application in classes or other teaching situations for any type of movement or physical activity course. The app could be used by both learners and instructors at the point of application to assist with the correct performance of a test or exercise.
